# Metastatic Spinal Cord Compression Secondary to Liver Cancer

**DOI:** 10.1155/2017/1679523

**Published:** 2017-11-02

**Authors:** Daniel Gams Massi, Japhari Nyassinde, Ngor Side Diagne, Ramy Abdennaji, Kamadore Toure, Moustapha Ndiaye, Amadou Gallo Diop, Mouhamadou Mansour Ndiaye

**Affiliations:** ^1^Department of Neurosciences, Fann National Teaching Hospital-Cheikh Anta Diop University, 10700 Dakar, Senegal; ^2^Department of Radiology, Fann National Teaching Hospital-Cheikh Anta Diop University, 10700 Dakar, Senegal; ^3^Health Sciences Unit, Thiès University, 221 Thiès, Senegal

## Abstract

Metastatic spinal cord compression (MSCC) is a medical emergency that requires rapid diagnosis and treatment to reduce pain, to preserve neurological functioning, and to prolong survival. The diagnosis of liver cancer is often neglected in the differential diagnosis of MSCC. Treatment is usually palliative and evolution is often fatal. This is a case report of a 28-year-old patient living in Mauritania hospitalized in November 2014 at the neurology department of Fann national teaching hospital in Dakar, for the management of a chronic dorsal spinal cord compression. The radiological and laboratory investigations done revealed the metastatic compression originating from a liver cancer with elevated alpha-fetoprotein and aspartate transaminase, positive hepatitis B surface antigen, and multiple metastasis in the lungs, mediastinum, ribs, iliac, and peritoneum. The hip joint X-ray showed a spontaneous fracture of the right femoral neck. The multidisciplinary treatment was palliative and the evolution was fatal within the month of hospitalization. Earlier diagnosis and treatment of MSCC may not have saved the life of the patient but may have prevented much suffering and would likely have prolonged the life of a young man.

## 1. Introduction

Metastatic spinal cord compression (MSCC) is a medical emergency, requiring rapid diagnosis and treatment. Described for the first time by Spiller in 1925, it consists of an extrinsic compression of the epidural space by a metastatic tumor [1]. In industrialized countries, with an aging population, MSCC is a common complication of cancer, particularly of cancer occurring at an elderly age such as cancer of the prostate, breast, and lung [2]. However, MSCC can occur at any age in virtually any type of cancer. Particularly in industrializing countries, physicians need to be aware of the different demography and different cancer epidemiology than that in industrialized countries, and they need to be aware that MSCC is more likely to occur in younger patients with uncommon tumors. Therefore, we wish to report this dramatic case of a young patient with MSCC from liver cancer. Few cases have been reported worldwide among which there is no case in Senegal.

## 2. Patient and Observation

**Figure 1:**
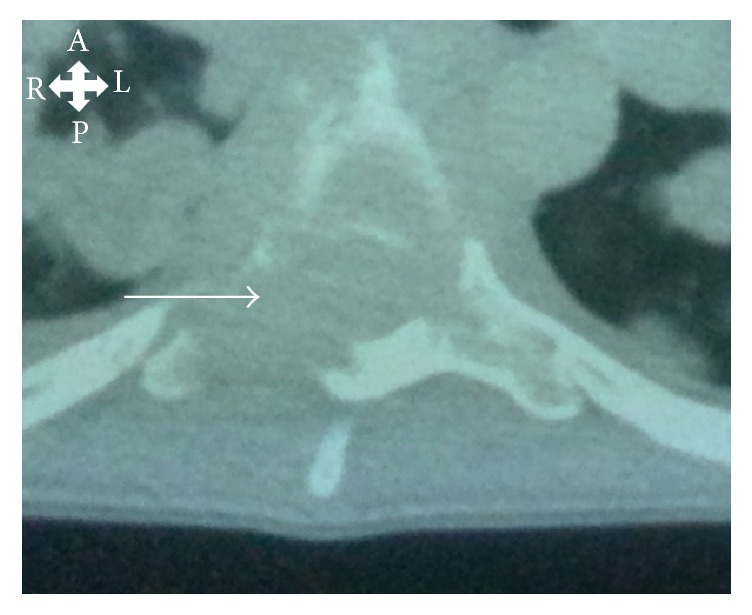
Thoracoabdominal CT scan (axial view) showing metastases located in thoracic vertebrae (white arrow). A (anterior), L (left), P (posterior), and R (right).

This is the case of a 28-year-old patient living in Mauritania with no medical history, hospitalized in November 2014 at the neurology department of Fann national teaching hospital in Dakar (Senegal), for a chronic dorsal pain and lower limbs progressive paralysis lasting for three months. On physical exam we found complete paraplegia, loss of sensation on both lower limbs to T6 level, loss of bladder and bowel control, jaundice, hepatomegaly, and weight loss. The diagnosis of spinal cord compression was made as well as the thorax, abdomen, and pelvic CT scan. The chest X-ray showed multiple pulmonary nodules of different sizes involving both lungs. The CT scan showed multiple hypodense and heterogenous masses of various sizes of the spinal cord at T6 and T9 and located in the lungs, mediastinum, ribs, iliac, and peritoneum and a hepatomegaly (Figure 1).

**Figure 2:**
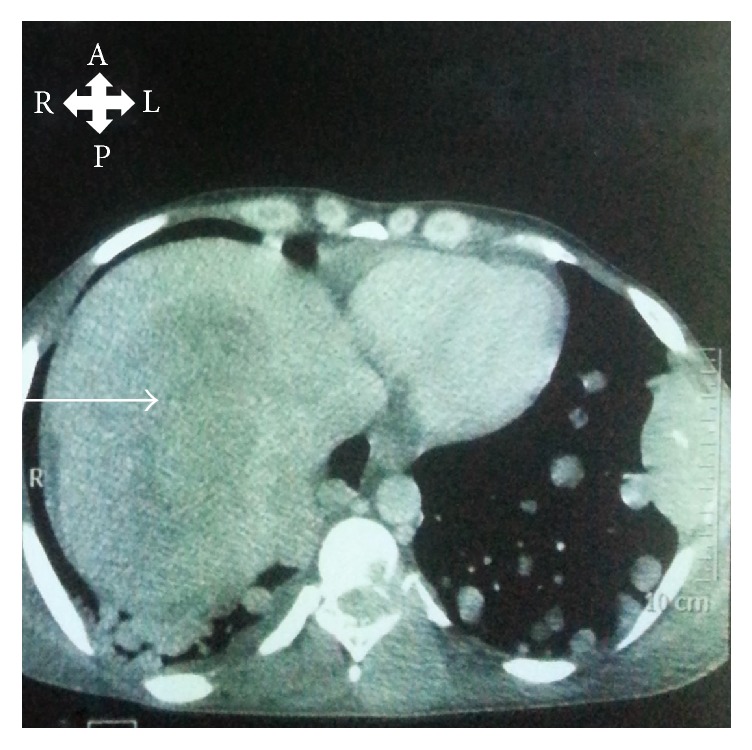
Thoracoabdominal CT scan (axial view) showing large heterogeneous and partially necrotic liver mass of 181 × 140 mm located in the right hepatic lobe (white arrow). A (anterior), L (left), R (right), and P (posterior).

This liver mass was large, heterogeneous, and partially necrotic and located in the right lobe (Figure 2). Elsewhere the lower limbs X-ray showed a spontaneous fracture of the right femur. Laboratory investigations demonstrated an elevated alpha-fetoprotein blood level (204.0 nanograms per milliliter) and aspartate transaminase (304 IU per liter), slightly normal alanine transaminase (40.8 IU per liter), positive hepatitis B surface antigen, a normochromic normocytic anemia (10 grams per deciliter), and low serum albumin level (32 grams per liter).

Despite the absence of a histological confirmation, the combination of clinical history, radiology, and biochemical markers justified a diagnosis of MSCC from primary liver cancer with extensive metastases. The treatment was multidisciplinary based on palliative measures. The patient received corticosteroids (methyl-prednisolone), pain killer (tramadol and morphine), and supportive care such as psychotherapy, physiotherapy, and nursing. The pain was partially controlled but there was no improvement of neurological signs. The evolution was fatal within a month of hospitalization.

## 3. Discussion

Metastatic spinal cord compression (MSCC) from primary liver cancer metastases is rare and represents less than 1% of secondary locations [3]. Liver cancer is the fifth most frequent cancer worldwide and has the third highest mortality [4]. Liver cancer is rare in industrialized countries, but is common in industrializing countries with a high prevalence of hepatitis B and hepatitis C and chronic alcohol consumption [5]. In Senegal, liver cancer is the first cause of cancer deaths in males, and third in females [4]. Prevention of hepatitis B is probably the most effective way to decrease incidence and reduce death. Alpha-fetoprotein is the most useful marker for the diagnosis of liver cancer and a highly elevated blood level is strongly suggestive even though a normal level does not completely rule out the diagnosis [4].

Hepatitis B surface antigen and tumor markers were also positive in our patient. Magnetic resonance imaging (MRI) is the gold standard for the diagnosis of spinal cord compression but is not widely available in large parts of the world. However, conventional X-rays and CAT scan were, at least in this case, sufficiently clear in showing the large vertebral metastases to corroborate the clinical diagnosis of MSCC [6]. The treatment of MSCC is often palliative including surgical decompression, bisphosphonates, corticosteroids, analgesics, radiation therapy, and chemotherapy [4, 6]. Although the vital prognosis of patients with MSCC is poor, rapid diagnosis and immediate palliative treatment are very effective in reducing pain in most patients and may revert or stop progression of neurological complaints [2, 6]. In patients with MSCC with a limited number of vertebral metastases, the combination of neurosurgery plus radiotherapy may prevent complete paralysis and improve survival [7].

## 4. Conclusion

We presented a case of metastatic spinal cord compression (MSCC) in a young adult with primary liver cancer. MSCC is a common complication of cancer in adults with more common cancers. This rare case illustrates that MSCC can occur at any age in virtually any cancer. Unlike Western countries, liver cancer is very common in countries with a young population and with a high prevalence of hepatitis B and hepatitis C, such as in Senegal. Since appropriate diagnostic and therapeutic techniques will be increasingly available, early diagnosis and immediate treatment of MSCC will be an increasing challenge, not only to reduce pain, but also to prevent neurological deterioration and to improve survival.
